# Advanced lithium-ion battery process manufacturing equipment for gigafactories: Past, present, and future perspectives

**DOI:** 10.1016/j.isci.2025.112691

**Published:** 2025-05-16

**Authors:** Atiyeh Nekahi, Ebrahim Feyzi, Muskan Srivastava, Firoozeh Yeganehdoust, Anil Kumar Madikere Raghunagtha Reddy, Karim Zaghib

**Affiliations:** 1 Department of Chemical and Materials Engineering, Concordia University, 1455 De Maisonneuve Boulevard West, Montreal, QC H3G 1M8, Canada

**Keywords:** Electrochemical energy storage, Energy engineering, Energy storage

## Abstract

Lithium-ion battery cell manufacturing depends on a few key raw materials and equipment manufacturers. Battery manufacturing faces global challenges and opportunities as various regions, including Asia, Europe, North America, and emerging markets, seek to scale gigafactory production and innovate equipment manufacturing pathways. Regions can enhance battery resilience by investing in advanced technologies, optimizing resource utilization, and adopting sustainable manufacturing practices. Investing in local technologies, taking advantage of green energy and processes, relying on local expertise and research opportunities, and strengthening partnerships among smaller suppliers are some solutions. Manufacturing equipment evaluation highlights significant challenges in electrode preparation, cell assembly, and finishing. Using space-saving machinery and cost-effective, scalable technologies that can adapt to new battery advancements is a practical solution. Continuous mixing of initial materials, double-sided coating, dry processes, electrolyte recovery, precise stacking, simultaneous formation in multiple cells, and online process validation accelerate the global shift toward electrification.

## Graphical abstract

**Figure:**
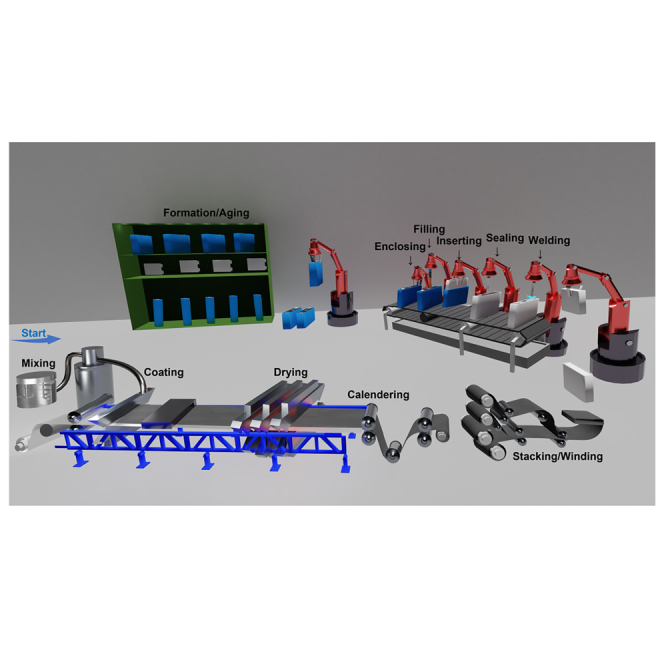

## Equipment for LIB manufacturing

### Manufacturing process

The global demand for Li-ion batteries (LIBs) has been increasing rapidly because of the popularity of electric vehicles (EVs) and energy storage. The transition to EVs drives this surge in demand as part of global efforts to address climate change, with many regions focusing on EVs to reduce greenhouse gas (GHG) emissions. This huge interest in the transition arises mostly from a dramatic decline in the real price of lithium-ion cells since their commercial introduction in 1991. This drop is attributed to factors such as the improved knowledge of LIBs, economies of scale, and advancements in battery chemistry.^1^

Lithium iron phosphate (LFP) has emerged as a cost-effective and safe option among various LIB chemistries. Between 2010 and 2020, prices dropped 77% from approximately $469/kWh to $102.5/kWh (all the prices in this paper are in $USD).^2^ As of 2020, pack-level prices totaled approximately $132/kWh, while cell-level prices were as low as $99/kWh.^3^ There was a modest price increase of around 8% in 2022 due to raw material costs, but projections still place prices in LFP at the level of $57.9/kWh (NCX scenario) or even $48.6/kWh by 2030 based on technological advancements and market trends.^4^ Advancements in battery chemistry by the development of LFP technologies and innovations in manufacturing, such as dry electrode processing, have mostly contributed to cost reductions and enhanced battery performance.^5^ By these facts, Europe, for example, has set a target to achieve GHG neutrality by 2050.^6^ As a result, the number of gigafactories is anticipated to increase, with a projection of ∼120–150 new battery production facilities globally by 2030.

The annual worldwide LIB demand will also grow from 700 to 4,700 GWh by 2030.^7^ While Asia continues to dominate production, regions across Europe, North America, and emerging markets in Africa and Latin America are scaling up gigafactory capacities and innovating new technologies. For example, India is expanding its battery manufacturing capacity with a focus on sustainable processes, while South America, led by countries like Chile, is leveraging its lithium reserves to support a globally integrated supply chain. Volkswagen Group’s subsidiary, PowerCo, has invested $48 million to establish a partnership with the Canadian lithium company Patriot Battery Metals. This collaboration aims to secure the lithium supply chain for PowerCo’s cell production facilities in North America and Europe.^8^ Toyota’s first global battery manufacturing plant in North Carolina^9^ and Asahi Kasei’s battery component plant in Port Colborne in Ontario^10^ are admired for North America’s attempt toward battery manufacturing. In addition to Europe and North America, Asia, particularly China and Japan, leads innovations in automated manufacturing processes and the integration of AI in battery manufacturing. In Asia, companies like BYD in China and LG Energy Solution in South Korea utilize AI to optimize production lines and reduce operational costs. India’s battery manufacturing industry is also growing, supported by the ₹18,100 crore ($2.4 billion) to establish 50 GWh of domestic production capacity. However, the industry faces challenges due to its 90% reliance on lithium imports from countries such as China, Australia, and Argentina, which raises concerns about raw material security.^11^

Despite the mentioned growing attempts to build up the gigafactories, many of them are currently operating below full capacity. This overcapacity can be attributed to several factors. First, many of these facilities are newly launched and scaling up requires time to optimize production lines and ensure quality control. Second, the shortage of raw materials such as lithium, nickel, or graphite restricts scaling up to full capacity. Third, uneven demand growth leads to localized oversupply in certain markets and short-term overcapacity. All these issues reflect production capacity and put it in a transitional period, which is hindering the battery industry’s rapid evolution.^12^

To remain competitive and operate at full capacity, battery producers must adopt technologies and solutions that ensure efficient, reliable, safe, and sustainable manufacturing practices.^13^ A reliable raw material supply chain, autonomous design in manufacturing, improved production line (in terms of cost, environmental aspects, performance, and longer life), and dealing with end-of-life (EOL) batteries must be considered. These are complicated issues all around the world.^14^ At the laboratory level, sophisticated cell technologies are utilized to design and evaluate electrodes, emphasizing slurry uniformity through different synthesis and preparation techniques, whether wet or dry. As the process transitions to industrial-scale production, many parameters must be meticulously regulated, including humidity, processing time and speed, the sequence of material additions, viscosity, coating, electrode loading, drying, electrolyte additives, and electrode cutting. Each of these elements is crucial for ensuring the final product’s efficiency, consistency, and quality, with electrode design and process optimization being key to realizing high power and energy density in the batteries.^15^^,^^16^ Despite lab-scale innovations, such battery development is not properly transferring to large-scale manufacturing because of the gap between academia and industry. Critical challenges of scaling lab developments to LIB industrial manufacturing regarding materials, processes, and cell designs must be properly resolved. This ensures the employment of techniques such as dry electrode coating to improve cost efficiency and performance.^17^ In addition to these features, the manufacturing process must undergo a significant transformation toward smart manufacturing. The production and assembly of battery electrodes directly impact the performance of these batteries, which necessitates improved production technologies for better results. Smart manufacturing, which integrates machines, objects, instruments, and human workers, is becoming increasingly common in the battery industry. This evolution enhances flexibility, customization, and collaboration among machines, optimizing the overall performance of manufacturing systems. Manufacturers can boost efficiency, quality, and adaptability by utilizing organized and interdependent machines. These modifications ultimately enable the scaling of battery production to meet the rising global demand.^18^^,^^19^^,^^20^

**Figure 1:**
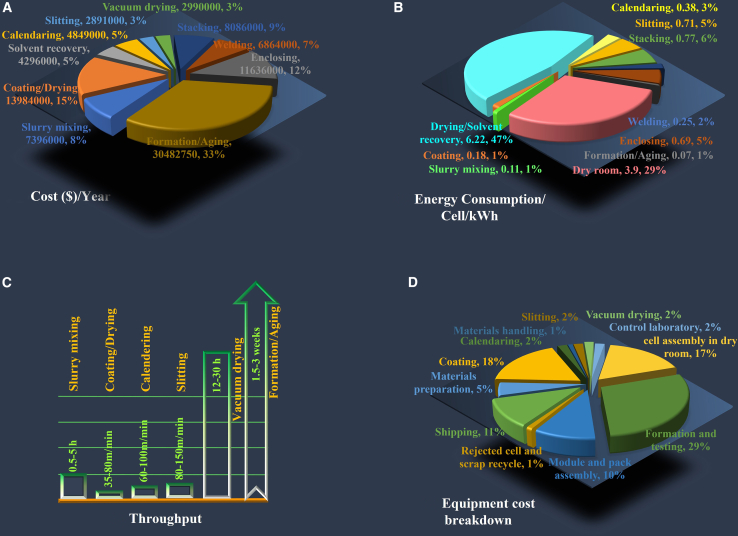
LIB manufacturing factors (A–C) LIB manufacturing process factors of (A) cost per year, (B) energy consumption per kWh cell, and (C) time consumption (data are not available or depend on the cell design for the steps that are not mentioned) for a plant of 67-Ah LiNi_0.6_Mn_0.2_Co_0.2_O_2_ (NMC622)/graphite cells with 100,000 EV battery packs/year capacity. Data from.^22^ (D) A breakdown of the installed capital equipment costs for the baseline plant, which has an annual production capacity of 100,000 EV batteries, for a total of 6.0 MWh per year. Data from.^24^

Finally, machine builders have a crucial role in the battery value chain, which can provide sustainable manufacturing practices through technological advancements,^21^ the main focus of this review paper. Although manufacturing contributes almost 25% of the LIB cost^22^ (the remaining 75% is attributed to the materials including cathode with 51%, anode with 12%, separator with 7%, electrolyte with 4% and others^23^), its progress has yet to be improved. Formation/aging (32.61%, amounting to 30,482,750 $/yr), coating/drying (14.96%, equating to 13,984,000 $/yr), and enclosing (12.45%, corresponding to 11,636,000 $/yr) revealed the biggest manufacturing cost share (Figure 1A).^24^ The energy use percentage is also illustrated in Figure 1B, with drying and the dry room sharing more than 75%. Formation/aging (up to 1.5–3 weeks) and vacuum drying (12–30 h) are the most time-consuming steps (Figure 1C). The data in this paper are related to the facilities typically designed for GWh–scale production (industrial–level, mass–production capacity). Most of the developments in battery production target GWh–scale production systems to meet the global LIB demand surge, especially in EV and stationary storage applications. The scale-up from MWh–scale (utilized principally for R&D or early validation) to GWh–scale presents new challenges in process control, speed, and integration that must be addressed through smart manufacturing and high–throughput design.

The technology choice in cell manufacturing can vary cell costs by 15–30%, a range of € 60–119 per kWh.^25^ Therefore, reconsidering the process, such as using solvent-free dry processes to eliminate drying,^26^ two-sided coating with simultaneous drying, and forming multiple cells simultaneously, would save time, cost, and energy. Considering the environmental aspects of meeting the net-zero scenario, closed-loop solvent recovery systems in battery coating processes are offered. For example, the achievement of 90% recovery of solvents from exhaust air streams by Dürr Group showcases the contribution of technology to eco-friendly manufacturing.^27^ A cost breakdown of the plant capital equipment is illustrated in Figure 1D, with the most significant expenses being formation cycling and testing, electrode coating, and cell assembly in the dry room.^24^ Considering these expenses, equipment builders must redesign machines and re-evaluate processes to offer solutions for these most expensive and time-consuming steps. Festo offers high-precision automation components for battery manufacturing with pneumatic and electric actuators, valves, and handling systems to improve electrode coating, cell assembly, and battery pack production efficiency.^28^ GROB-WERKE GmbH & Co. KG developed advanced automation systems utilizing precision robotics and modular production lines for assembling battery modules and packs.^29^ Körber AG’s cell maker technology provides high-speed, fully automated battery cell production featuring precise material handling and laser welding to ensure consistent quality.^30^ Eirich Machines Inc. offers MixSolver technology to reduce energy consumption in electrode slurry production. It requires approximately 400 kWh for 20 m^3^ of slurry compared to 8,000 kWh used by traditional planetary mixers, with 95% energy reduction.^31^ Jagenberg Converting Solutions GmbH revealed high-precision slot die design and diffusion-optimized drying technologies to improve both efficiency and quality in battery electrode production lines.^32^ PEC offers comprehensive solutions for cell activation processes of electrolyte filling, formation, and aging. They employ precision dosing systems and controlled environmental chambers to ensure optimal electrochemical performance and maintain the safety of battery cells.^33^ Together, these companies have made significant advancements in battery technology through their specialized equipment and technical innovations.

### Manufacturing pathways

**Figure 2:**
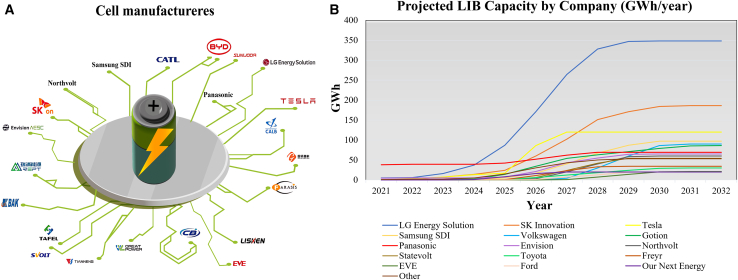
Cell manufacturing projection (A) Global cell manufacturers with >$1 billion market value. Data from.^34^ (B) Projected LIB capacity (GWh/year) by company in North America. Data from.^35^

Giant machine manufacturers for LIB lines are trying to advance the technology and overcome the abovementioned challenges (Table 1) for cell manufacturing companies (Figure 2A).

**Table 1:** Battery equipment manufacturers

Supplier	Origin	Establishment	Mixing	Coating	Calendering	Slitting	Stacking	Winding	Welding	Sealing	Filling	Forming	Testing
LEAD	China	1999											
Manz AG	Germany	1987											
Hitachi High-Tech	Japan	1947											
Sovema	Italy	1967											
Xiamen Tmax	China	1995											
Yinghe	China	2006											
Xiamen ACEY	China	2009											
Dürr Group	Germany	1895											
Hosokawa	Japan	1923											
Schuler	Germany	1839											
Netzsch	Germany	1873											
Hirano Tecseed	Japan	1951											
IPG Photonics	US	1990											
NEWARE	China	1998											
Hana Technologies	South Korea	1985											
PNT (People and Technology Inc.)	South Korea	2003											
Hirata	Japan	1951											

Data are drawn from each manufacturer’s website.

Cell manufacturers play a key role in the battery value chain, especially in midstream (like electrode processing and cell assembly) and downstream (such as module and pack integration) stages. While they do not set prices directly, their scale and technical demands influence supply terms, product specs, and how fast others in the chain can adapt. These cell and EV manufacturers are CATL (36.7%), BYD (15.8%), LG Energy Solution (13.8%), Panasonic (6.8%), SK Innovation (5.1%), CALB (4.7%), Samsung SDI (4.6%), Gotion (2.4%), EVE (2.1%), SUNWOOR Energy (1.4%), and others (6.6%), which are primarily located in Asia. Recorded data for gigafactory plants also show the most significant contribution of Asia: 2,691, 2,293.5, 1,897, and 1,564 GWh of capacity are placed in Greater Asia, China, Europe, and North America, respectively.^34^

Figure 2B shows the top LIB producers. LG Energy Solution (300 GWh) and SK Innovation (180 GWh) lead in the projected capacity by 2028. The Gigafactory capacity figures mentioned in the figure include both commissioned capacity and announced (planned/future) capacity (future projections for 2028 and beyond). Panasonic currently operates at the largest capacity at Tesla’s Gigafactory and is trying to increase its capacity. Tesla is expanding Nevada’s gigafactory by 100 GWh and is planning a similar project in Austin. Samsung, Volkswagen, and Gotion each target approximately 100 GWh by 2032.^35^ As the primary equipment purchasers, these cell producers significantly affect the manufacturers (Table 1) based on their needs. The expected battery cell market growth from €20 billion to €550 billion within a decade adds more pressure. Although equipment manufacturers could benefit from this growth, they must aim for a 50% compound annual growth rate (CAGR) or at least a 35% CAGR to maintain their market share.^25^

**Figure 3:**
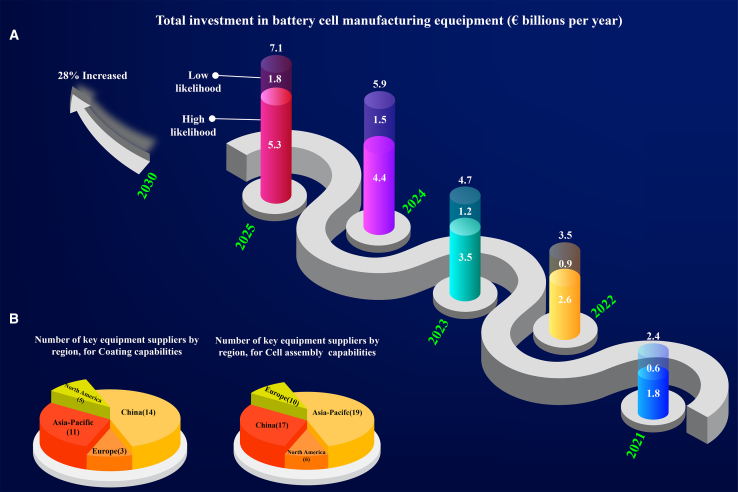
Investment on equipment and key suppliers. (A) Total investment in cell manufacturing equipment is increasing to €5–7 billion by 2025. (B) Key equipment suppliers for coating and cell assembly by region. Data from.^7^

The giant equipment manufacturers (Table 1) are located in Asia, similar to major cell manufacturers, with significant shares from China (14% in coating and 17% in cell assembly), Japan, and Korea (11% in coating and 19% in cell assembly for Asia-Pacific). Europe (3% in coating and 10% in cell assembly) and North America (5% in coating and 6% in cell assembly) follow (Figure 3).^7^ Battery manufacturing globally faces the challenge of reliance on equipment. This concern pushes regions worldwide, including Asia, Europe, North America, and emerging markets, to innovate equipment manufacturing pathways. Each region contributes technological advancements to optimize production and meet growing global demand. However, industrialization and machine production need multi-professional teams and training of operators as well as mechanical, computer, electrical, and chemical engineers. Collaborative efforts between industry and academia will be key in overcoming technological challenges. They must emphasize a range of strategic initiatives, such as setting up regulations, investing in original equipment manufacturers (OEMs), supporting innovations and expertise, demanding geopolitics and sustainability, and relying on green energy and processes. To foster a competitive and sustainable global battery manufacturing industry, all regions are pushing for technological innovation. To this aim, they focus on building a more sustainable, competitive industry that aligns with global ethical and environmental standards. China’s investment in circular economy practices by developing recycling technologies and India’s efforts in using solar energy for manufacturing are some examples. Africa and South America are also exploring ways to reduce carbon footprints through localized supply chains and renewable energy.

### New equipment markets

The International Energy Agency admits the necessity of new OEMs to provide affordable options with competitive prices, enabling EV’s mass adoption.^36^ Europe is working to take a significant part in the manufacturing realm. Established battery cell companies and startups plan to build up to 965 GWh of production capacity in Europe by 2030, which is 28% of the global target of 3,500 GWh, to support European EV manufacturing. Meeting this demand, 20 times increase from 2020, will require approximately 30 new battery manufacturing facilities across Europe, with up to €100 billion in capital expenditures (Figure 3A). Approximately 60% of this investment will go to battery cell manufacturing equipment, creating a €5–7 billion opportunity for Europe’s manufacturing equipment industry by 2025.^7^ Stellantis and CATL have formed a joint venture with a €4.1 billion investment to develop a large-scale LFP battery plant in Spain with a target capacity of up to 50 GWh. CATL already has two plants in Germany and Hungary, and this joint venture will further strengthen CATL’s collaboration with Stellantis in delivering cost-effective batteries for EVs in Europe.^37^ BMW Group has formed a long-term partnership with Rimac Technology to develop and produce high-voltage battery technology.^38^ Such alliances are essential in strengthening Europe’s share in the EV market. Figure 3B shows the shares of various continents in coating and assembly equipment manufacturing.

**Figure 4:**
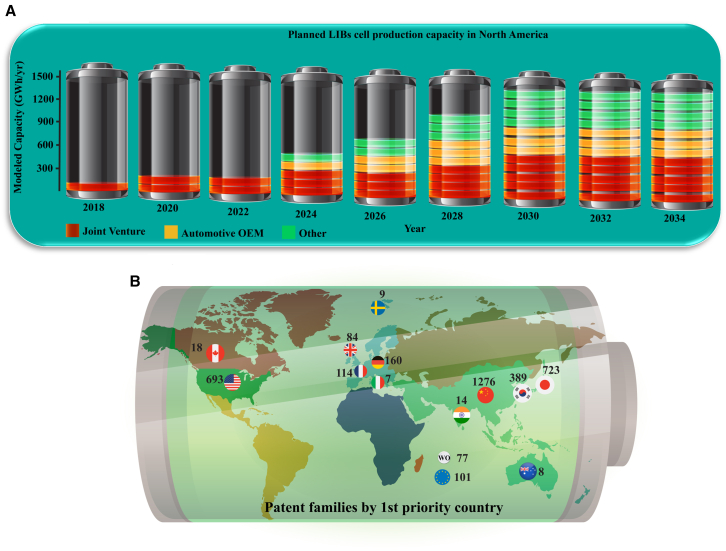
Cell production and the potentials (A) Projected capacity of lithium-ion cell production in North America from 2018 to 2035 categorized by plant ownership.^25^ (B) The number of patent families based on the first priority country for “lithium-ion battery cell manufacture” keywords in 20 years until the end of 2022 (patents have not yet been prioritized in many countries after this date). Based on the data obtained from the World Intellectual Property Organization (WIPO).^39^

Figure 4A examines the US companies that have invested in LIB cell production. The largest share of the projected capacity, approximately 580 GWh, comes from joint ventures between energy companies and traditional automakers. Another 520 GWh of battery capacity is planned by battery companies operating independently of automakers. The remaining 260 GWh of investments comes from traditional automotive OEMs, including Ford, Tesla, and Volkswagen, which have opted to control cell production for some of their EVs. Notably, Tesla, also identified as an energy company, allocates some of its manufacturing capacity to stationary energy storage.^35^

Nations must strengthen local manufacturing, reduce reliance on imports from others, and gain better control over their essential resources and equipment. However, each one of these solutions may cause some issues. For example, reducing imports poses a challenge for newcomers, particularly in areas where critical battery metals are scarce. Without access to local raw materials, the reliance on imports remains significant. However, investing in cell manufacturing can still be beneficial if backed by robust recycling initiatives, strategic partnerships, or advanced battery chemistries that minimize dependence on limited resources.^40^^,^^41^^,^^42^ In addition, newcomers encounter considerable challenges when competing with established battery suppliers, primarily due to high capital costs, supply chain management, and technological expertise. However, competition can arise through innovative chemistries, cost-effective manufacturing processes, or government incentives. Established suppliers may prefer collaboration with newcomers if it benefits their supply chain, such as by securing raw materials, accessing new markets, or advancing next-generation technologies.

Regions across Asia, Europe, North America, and emerging markets focus on expanding gigafactory production. This includes not only securing raw materials but also leveraging local technological advancements. For instance, while China leads in large-scale production, countries like India are accelerating efforts in local cell manufacturing, and South American nations like Chile, with their lithium reserves, are exploring partnerships to localize production and integrate renewable energy sources. Government support, such as tax incentives and subsidies for domestic manufacturers, could encourage local production. Investing in OEMs is a promising approach. With solid engineering and research expertise, regions can develop advanced battery production equipment. The number of prioritized patents in cell manufacturing innovation over the past 20 years is shown in Figure 4B (Based on the data obtained from the World Intellectual Property Organization (WIPO)^39^), with China (34%), Japan (20%), the US (19%), and Korea (10%) taking the lead. By relying on these innovations and collaborating with established battery equipment manufacturers, OEMs can grow and create a more globally balanced production network.

A strong approach for developing equipment manufacturing is for companies to address machinery gaps by acquiring or collaborating with established battery equipment manufacturers. To create a viable hub, collaboration among cell manufacturers, equipment suppliers, and public entities is crucial. Without cooperation and new global partnerships among stakeholders, cell OEMs, and large equipment manufacturers, over 90% of the market will likely be maintained by the present suppliers. This collaboration could bring rewards of up to €300 billion by 2030 and could help in addressing equipment shortages. For example, Tesla agreed with Grohmann Engineering to secure equipment supply for its gigafactories.^25^ Similarly, Manz AG and China’s Shenzhen Yinghe Technology Co., Ltd. (Yinghe) teamed up to improve market access in Europe, China, and the United States. An emerging trend is building manufacturing plants closer to demand centers, such as Kore Power in the United States. Another trend is for Chinese battery companies, such as CATL, to establish and expand their manufacturing in other continents, particularly Europe.^43^ Manz AG, GROB-WERKE GmbH & Co. KG, and Dürr Group have entered a strategic partnership to jointly acquire and execute projects for outfitting complete battery manufacturing plants. They aim to capitalize on LIB production technology’s significant growth opportunities and encompass the entire value chain. Through this collaboration, they aim to establish themselves as a European system provider for battery production facilities, offering an alternative to predominant suppliers. The trio seeks to set new, innovative machinery standards “made in Europe”.^44^

Greener processes and sustainable energy resources are advantages for new manufacturers compared with the traditional counterparts. Although both established suppliers and new entrants in the battery industry are pursuing greener processes, newcomers emphasize on the fact because of the benefit from government incentives for sustainable practices. They can focus on ecofriendly manufacturing in machinery buildings, including solvent-free processes, energy-efficient equipment, and green energy, such as hydroelectricity, solar, and wind. Strict environmental regulations and restricted ethical production ensure to meet global climate goals, which appeals to companies and consumers who are increasingly concerned regarding sustainability. Overall, sustainability is a growing trend driven by the force of regulations, consumer demand, and economic incentives.

**Figure 5:**
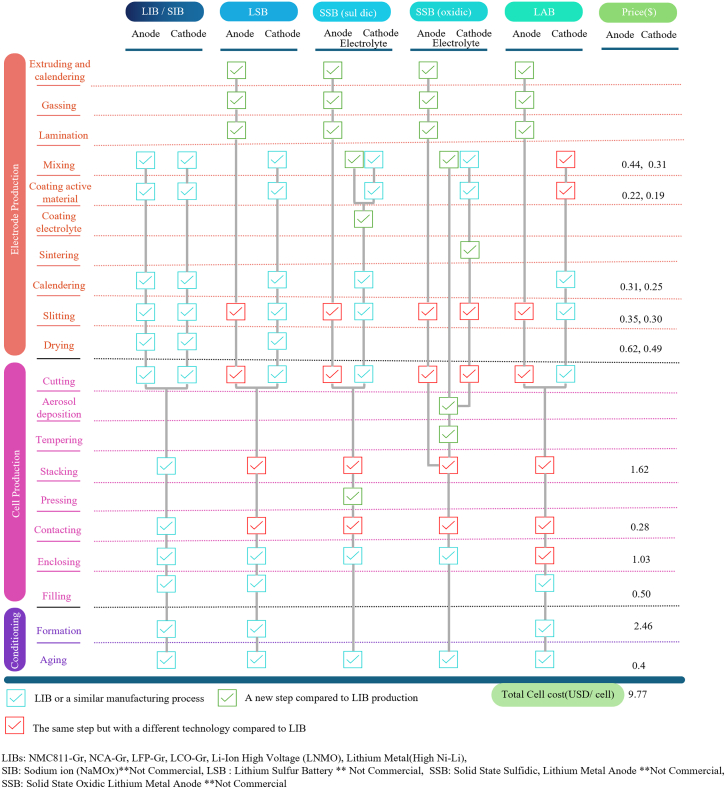
Manufacturing differences for the critical battery chemistries and the forecast for novel battery systems Reproduced from.^47^^,^^48^

New pathways can also emphasize the fast-changing battery technology and the invention of different chemistries or various battery systems, such as LFP and lithium iron manganese phosphate (LMFP) chemistries, sodium-ion batteries, sulfur batteries, and solid-state batteries.^45^^,^^46^^,^^47^ With the growing demand in the EV market, such post-lithium-ion technologies have been more appealing. To speed up their adoption, the compatibility of these new technologies with the current infrastructure must be considered in terms of material chemistry, cell design, and production steps.^48^ Post-LIBs production generally requires less energy than LIBs, with energy demand varying based on cell chemistry and density. Advancements in production technology could cut energy use by up to 66% by 2040, which can significantly reduce GHG emissions. The study highlighted the need to optimize production processes and materials to lower costs and environmental impact while supporting the growing battery market.^6^ With all the modifications in the manufacturing in addition to the cell developments, leading EV manufacturers such as Ford and Renault expect cell costs to reach around $47/kWh and $60/kWh by 2030. In a separate analysis, LIB cell production cost is estimated to be approximately $90/kWh by that same year. This study projects 2030 cell costs as low as $48.6/kWh under scenarios where LFP chemistries dominate the market.^4^ Reaching adaptable technologies to accommodate these inventions is vital (Figure 5). Otherwise, the entire system must be changed with an extraordinary consumption of time and credits, hindering industries’ tendency to pursue technological advancements. The recent challenge of replacing lithium with sodium seems promising because of their similarities in some niche markets. It has attracted significant attention from research and industrial partners only because of the possible capabilities of redesigning and adjusting the present manufacturing processes.

## Equipment for electrode preparation

### Premixing

Large-scale electrode preparation in battery cell manufacturing plants starts with pregrinding and premixing, two essential steps for higher yields in the downstream mixing and coating steps.^49^ These processes offer advantages for a cost-effective energy-saving mixing process with a more even distribution of mixed slurry materials. The pregrinding unit reduces the premixing time by up to 50% and the cleaning effort for large batches. It results in stable rheological properties of the ground products without sedimentation after predispersion. Subsequently, energy consumption is reduced by up to 30%, and the premixing time is lowered by up to 50% (the ProPhi pregrinding unit offered by NETZSCH Group).^49^ A precursor and active material (AM) co-grinding process is also proposed for the proper AM preparation as a basis for high-performance LIBs. Thus, the desired particle size is achieved and the slurry is optimized for downstream processing (proposed by Bühler).^50^

Premixing is performed in separate mixers for combinations of AMs, e.g., LFP, C, and VGCF mix using a mixer and polyvinylidene fluoride (PVDF) and NMC mix using another. Then, these two premixed materials are passed into high-shear mixers for the primary mixing process. These components are initially dry-mixed and then subjected to a brief mechanical grinding process. This step ensures uniform dispersion and intimate contact among the powders before the electrode fabrication begins. As a result, this pre-treatment enhances homogeneity and improves interfacial contact, which contributes to better electronic conductivity and mechanical integrity of the electrode. In contrast, the conventional electrode fabrication process entails dispersing these individual components directly into a solvent (commonly N-methyl-2-pyrrolidone, NMP) without any prior dry mixing or grinding. This method often requires prolonged stirring and sonication to achieve uniform dispersion, which consumes more energy and time. Additionally, it may still result in agglomerates or phase separation during the slurry casting process. The premixing and pregrinding approaches present a cost-effective and energy-saving alternative. It reduces the reliance on prolonged solvent–based dispersion and decreases the overall processing time and energy input required during slurry preparation.^51^^,^^52^

Mechano Fusion utilizes intense mechanical energy to trigger mechanochemical reactions between particles, which results in new particle properties. It can even control particle shape (rounding and flattening). Rounding is desired for anode materials to achieve a higher filling rate and sintering performance. Mechano Fusion (a Hosokawa Micron Corporation registered trademark in Japan) is an example of how manufacturers are addressing the need for consistent particle distribution and reduced processing times. Unlike wet particle composing systems, which are limited in the types of processed materials and drying requirements, Mechano Fusion offers a simpler mechanical process that expands the range of possible combinations without the metal or ceramic balls to avoid any side reactions. Beyond surface fusion, the system also enables advanced particle surface control and achieves a higher degree of material mixing than traditional powder mixers.^53^

### Mixing

**Figure 6:**
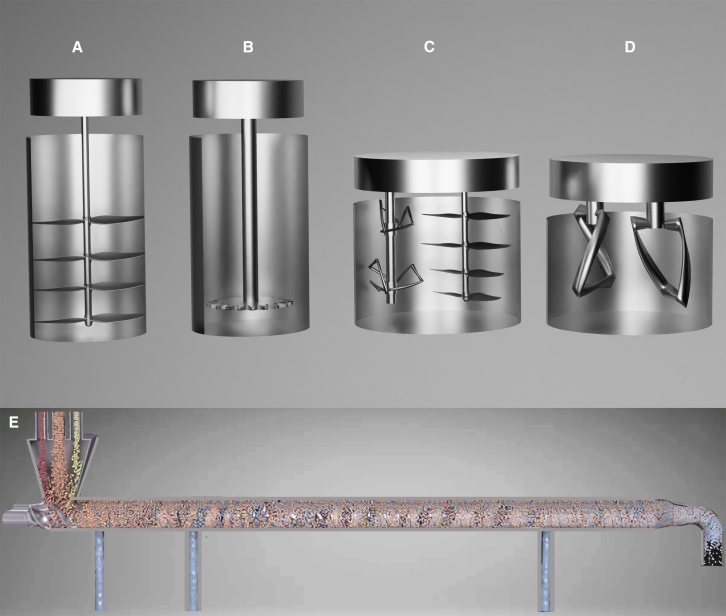
Current mixing technologies (A) Schematic of (A) simple hydrodynamic shear mixing as a widely used slurry mixing method in battery manufacturing. (B) NETZSCH Group’s intensive mixer (PMD-Mixer) is a planetary rotating disk that moves vertically to disperse and homogenize materials. (C) NETZSCH Group’s PMH/PML mixing and kneading machine with a dual rotational motion. (D) INOUE planetary mixer featuring a pair of novel-designed open-frame blades. (E) Bühler’s continuous twin-screw mixer for constant mixing in large-scale battery production plants. The schematics are produced based on the original mixers from the manufacturer’s websites.

Slurries are made by mixing AMs, binders, and solvents from the previous premixing process. The different combinations among those three highly affect coating and drying. Hydrodynamic shear mixing (Figure 6A) is the most common method used in battery manufacturing for slurry mixing due to its cost-effectiveness and scalability. Shear forces break down particle clumps, making an even distribution of AMs and binders in the slurry. However, more uniform slurries are needed with a more time-consuming, energy-saving process. Various mixer manufacturers have come up with solutions to overcome these challenges. Intensive mixing technology has improved the efficiency of battery mass production by enabling more homogeneous mixing with reduced energy consumption. Advanced equipment, such as the PMD-Mixer, has been developed to meet the growing needs of battery manufacturers (Figure 6B). The disk rotates to disperse, homogenize, and wet materials and moves vertically to disperse the materials more evenly. The PMH/PML mixing and kneading machines (Figure 6C), proposed by NETZSCH, use a planetary system with two mixing elements rotating on a central axis in a stationary tank while also rotating on their axes. This dual rotational motion covers the entire mixing area and delivers optimal dispersion. At the same time, the rotating wall/floor scrapers enhance mixing and kneading while providing efficient heat transfer to the tank walls.^49^

A platform is designed to enhance the mixing equipment using a planetary mill in the mixing process. This mixer, Dreamline 2.0, designed by LG Energy Solution in Ultium Cells plants, a joint venture with General Motors, upgrades the capacity from the current 2,300 L to 3,000 L.^54^ Another mixer is a planetary one that features a pair of novel-designed open-frame blades; each rotates on its own axis and follows a planetary orbit (Figure 6D). The precisely engineered clearance between the blades and the tank’s inner walls generates a powerful shearing force for superior mixing, kneading, and dispersion, proposed by INOUE.^55^ The mixing sequence significantly affects the electrodes’ rheological, mechanical, and electrochemical properties. Mixing carbon black (CB) with PVDF solution first creates a gel-like slurry that maintains its structure when NMC particles are added, forming porous clusters after drying, which enhances rate capability. Conversely, dry mixing CB and NMC leads to a dense PVDF and CB layer on NMC particles, offering higher cohesive strength but potentially hindering ionic transport and weakening the electronic connection.^56^ Finally, the mixed products in the mixers are transferred to a tank using a robot and then are passed to the coating step via the pipe.

Another crucial focus is shifting the wet mixing process from a batch to a continuous method. In this context, extrusion offers a promising approach for wet mixing because it produces slurries with less solvent. This strategy simplifies the process, reduces factory footprint due to higher productivity, lowers energy consumption, and minimizes waste.^57^ A continuous electrode slurry production process is designed for large-scale LIB manufacturing using a twin-screw mixer that integrates raw material dosing, premixing, kneading, fine-dispersing, and degassing into one system (Figure 6E). The invention of this continuous mixing is called a historic moment, a revolution by Bühler. A significant portion of the mixing is handled by the constant dosing of raw materials, resulting in a residence time of under 1 min in the mixer. With a production capacity of up to 2,500 L/h, continuous mixing can replace several batch mixers with a single, fully automated line, substantially reducing investment costs for large-scale production. This method requires thrice less specific energy than batch mixers to achieve the same product quality. In addition, the smaller system footprint reduces the dry room volume, cutting energy consumption for climate control. The high level of automation further decreases manpower workforce needs by 50%.^50^ Deaeration is typically performed after mixing because air or gas pockets formed during the handling of liquid or highly viscous products can create issues. Oxygen in the air can cause oxidation, spoiling fats or oils, or discoloration. For materials intended for coating, air pockets can result in porous or uneven surfaces. By contrast, deaerated products are more chemically stable and durable.^49^

With the increased interest in dry processes by battery manufacturers, dry mixing is becoming more popular. The dry mixing process for electrodes involves combining AMs, conductive agents, and binders in a solid state. Similar to slurry mixing, the dry method has uniform distribution challenges, which disrupt electron- and ion-transport pathways, increase internal resistance, and affect charge-storage capacity and stability. Advanced hot melt, secondary, or specific mixing techniques improve homogeneity. Binder type and size and particle size also significantly affect the uniformity and quality of the electrode. Premixing and solvent-free methods, such as electrostatic spraying or hot pressing, increase uniformity and performance in dry electrode fabrication.^58^ High-shear mixing enhances slurry quality in wet mixing, and extrusion is most effective for dry mixing. The minimum mixing volume, powder-to-solvent ratio, and viscosity are key factors to be optimized. The main challenge in extrusion is polymer breakage chain order, which decreases conductivity and mechanical properties. Sulfide-based SSBs struggle with solvents for dispersing AMs and binders, which makes dry methods difficult. Oxide-based SSBs require high shear rates. However, dispersion homogeneity is poor and limits interface quality, and sintering restricts the use of polymeric binders. Hybrid SSBs need premixing, and gel-based SSBs often rely on magnetic stirring, which complicates scaling. Mechanical stirring is commonly used for its simplicity and cost efficiency, but new, more energy-efficient agitators with continuous monitoring are being developed to reduce material waste and equipment costs. These advancements also demand new skills in the battery industry workforce.^57^ In addition, introducing a uniformly distributed binder that functions as a lubricant during dry electrode manufacturing in a roll mill improved shearing and expanded the processability window.^59^ Innovations in mixing technologies, i.e., high shear mixing or mechanofusion, offer substantial reductions in cost, energy consumption, and processing time. As an example, advanced mixing systems can reduce overall electrode slurry preparation costs by 15–30% due to lower maintenance, reduced solvent loss, and improved material utilization.^22^ Innovations like mechanofusion or continuous dry mixing have demonstrated extensive energy savings by eliminating high solvent evaporation and reducing mixing durations. A 46% energy saving was reported for the electrode drying and solvent recovery during LIB manufacturing.^60^

The viscosity of a slurry is characterized using a rheometer by measuring the flow and deformation properties of the materials. A sample is positioned between two plates or within a cylindrical measuring system, such as a cone and plate setup, and exposed to a rotating shear force. The induced shear stress determines essential rheological parameters, such as viscosity, elasticity, and yield point.^49^^,^^61^

### Coating

**Figure 7:**
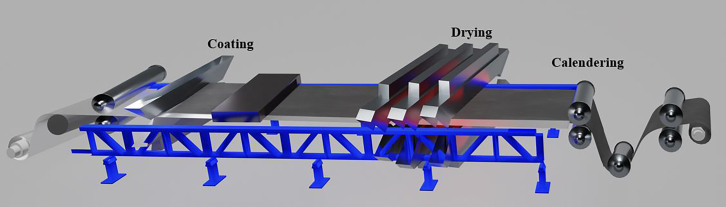
Schematic of electrode preparation, including double-side coating, drying, and calendering on the current collector foil

**Figure 8:**
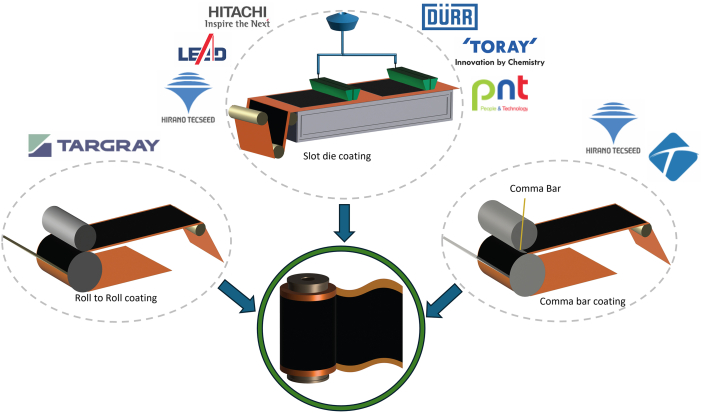
Three commonly used coating techniques: slot-die, roll-to-roll, and comma bar coating methods

After preparing the materials in the form of slurry paste, the three main techniques of coating, drying, and calendering are performed for preparing the electrode films on the current collector foil (Figure 7). The growing battery market is also dragging the coating machine market along with it at a CAGR of 15.5% and is expected to reach $5.76 billion by 2030.^62^ An in-depth observation of coating is vital because any variations in the coating microstructure or the slurry distribution can significantly affect the cell performance.^63^ Any nonuniformity in the coating impairs Li-ion transport and negatively affects the electrochemical performance of the cell. The viscosity of the slurry is a crucial parameter of the powder dispersion within the slurry. Various techniques are available for lab-scale coating, including spray coating, spin coating, dip coating, comma bar coating, ink-jet printing, electrophoretic deposition, doctor blading, and slot-die coating.^64^ Slot-die, roll-to-roll, and comma bar techniques are extensively used in industries (Figure 8). Leading manufacturers of electrode coating machines globally include Lead Intelligent Equipment Co., Ltd. (LEAD), Yinghe, Dürr Group, and Hirano Tecseed Co., Ltd., Hitachi High-Tech Corporation, PNT (People and Technology Inc.), and Xiamen Tmax Battery Equipments Ltd. (Table 1). These manufacturers offer products that meet the current needs of the battery manufacturing industries.

Depending on the technology, the coating process can either be pre-metered or self-metered. Slot-die coating is a pre-metered method, which means that the flow rate pumped to the coating station for a specific coating thickness is measured beforehand.^65^^,^^66^ This technique was invented by A.E. Begiun at Eastman Kodak Co. in 1954 to develop a precise, high-speed, and minimum waste coating method.^67^ In slot-die coating, the slurry is pumped into the cavity chamber or gap of the slot die, and as the foil moves beneath it, the gap between the slot die and the coil is filled with electrode slurry in the form of a coating bead.^68^ There are two commonly used slot-die coating techniques: back roller slot-die coating and free-span or tensioned-web over slot-die coating. In the tensioned-web method, the free tension generated by the web roller redistributes the coating and aids in achieving a uniform coating.^69^ Coating speed, volumetric flow rate, and web tension are essential parameters that define the quality of the wet film.^70^ Industrial slot-die coating machines can operate at speeds of up to 300 ft/min. However, this technique can produce uneven or heavy edges that cause challenges in the cell assembly stage.^68^

Comma bars and roll-to-roll coaters are other commonly used coating methods. Unlike the slot-die technique, these methods fall into the self-metered technique category. As the name suggests, the devices control the coating thickness rather than being predefined.^66^ The gap between the comma bars or roll bars and the back rolls allows the slurry to be coated on the electrode foil. Targray is one of the manufacturers of roll-to-roll coaters for R&D purposes with integrated drying equipment.^71^ A modification to roll-to-roll coater is known as comma reverse, where a rubber roll attached to the comma bar allows intermittent foil coating.^72^ The current design of batteries favors intermittent coating, which gives comma reverse technology an edge over other technologies. Intermittent coating can also be achieved using the slot-die technique using a pulsating action for dispensing slurry.^73^

Electrode slurry is coated on both sides of the current collector using single- or double-sided coating methods. In single-sided coating, two sides of the collector foil are coated and dried. The second slot die is positioned based on the dried coating thickness of the first side to ensure uniform coating.^69^ In 2011, a two-sided coating technique was patented,^74^ where two coating heads simultaneously coat both sides of the substrate. The double-sided coating offers increased coating efficiency and reduced processing time. These advantages proposed by Toshiba Corporation have been interesting to companies such as Dürr Group^27^ and Hirano Tecseed Co., Ltd.^75^ Recent advances in coating technology enable precise control of coat weight (in the range of 70–300 gm^−2^ dry) with sharp edges less than 3 mm, which improve manufacturing efficiency. Manufacturers such as Manz AG and Dürr Group offer solutions that meet these advanced requirements.^76^ Slot-die coating is commonly used in simultaneous double-sided coating, and researchers continuously work to make this process more efficient and advantageous.^77^ Dürr Group provides an integrated electrode manufacturing skid unit that includes coating, drying, solvent recovery, and distillation. This system achieves a 99% NMP recovery rate and 95% reusable solvent after distillation, with cost savings of over $2/kg while optimizing space efficiency. Dry electrode coating technology has significant advantages over traditional methods by eliminating harmful solvents like NMP. The dry electrode process removes the need for drying and solvent recovery and reduces the processing time by 21.6%. Furthermore, eliminating costs associated with those processes can lower overall electrode manufacturing costs by 10–15% while reducing energy consumption by 46%.^27^

As the demand for increasing energy density in batteries grows, one solution is increasing the thickness of the electrodes.^78^ The current electrode coating thickness ranges from 50 to 300 μm, but thicker electrodes with 40–50 mg/cm^2^ loading and more than 700 μm coating thicknesses are becoming more desirable. The gap between the foil and slot die must be increased to achieve this coating thickness. However, there is a limitation to the gap width because a large gap can cause an overspill upstream of the slot die, resulting in uneven coating. It is possible to widen the gap by varying the slurry content or modifying the slot-die structure, but adhering to the coating window is crucial. Confining the coating window for thicker electrodes can challenge machine manufacturers.^69^

Coating defects such as agglomerates, pinholes, metal particle contaminants, and nonuniform coating are other problems faced during this process.^79^ Beta transmission and charge-coupled device (CCD) camera inspection are the current technologies used in industries to detect coating defects such as pinholes, agglomerates, nonuniform coatings, and metal contamination in the electrode manufacturing process.^80^ During offline material testing, other methods, such as Raman spectroscopy, have also been used to detect differences in coating composition. These methods have high resolution to identify defects and prevent faulty cell construction.^81^^,^^82^ Laser calipers have been used to measure the wet thickness of the coating along a line scan as a substitute for the radiation-prone and costly beta transmission technique. The laser caliper system is positioned on the coater in an orthogonal manner to the substrate to ensure alignment even when the electrode thickness varies. For in-line laser measurement, a precision of <±2% was attained.^83^ IR thermography can efficiently identify in-line on a high-speed electrode coating system. This system consists of an IR camera through a series of mirrors pointed at the electrode exiting the hot drying oven. The camera records and analyzes the thermal radiation emitted by the coating. Because electrode coatings are solid materials with high thermal conductivity, heat must be transferred from the coating to the surface through pore-based convection and internal conduction. Defects in the coating often alter the thermal response.^79^^,^^84^

The use of such in-line detectors can help identify coating defects.^85^ The Smart Coaters are equipped with automatic coating thickness control and fully automated parameter configurations, such as the one proposed by Toray Engineering Co., Ltd.^86^ In-line detectors offer real-time surface density inspection to detect any abnormality observed during this process.^87^ This, in turn, helps in detecting variations in coating thickness, ensuring uniformity. Digital twin technology^88^ gathers and assesses in-line data from machines to achieve high-quality coating. Digital transformation of battery manufacturing equipment focuses on the standardization of battery coating machines, market time reduction, and profit enhancement. The project aims to create digital twins for efficient mass production systems in a collaboration between Siemens with Hirano Tecseed Co., Ltd.^89^

### Drying

In the battery manufacturing process, coating is followed by drying. The main purpose of this process is to remove the solvent used in the preparation of electrode slurry. Thus, it is an expensive and energy-intensive process. Typically, coating and drying processes occur in synergy. With the help of rollers, the coated foil is passed through the furnace and is heated continuously, ensuring that coating and drying occur simultaneously.^90^ Drying is a complex process that involves heat and mass transfer, with mass transfer being the controlling step.^63^ During drying, three main phenomena occur: solvent evaporation, diffusion of the binder, and sedimentation of particles. This process influences the electrode’s microstructure and its electrochemical and mechanical properties.^70^

Drying is not a single-step process. The liquid film shrinks, the liquid is removed from the surface, and pore emptying begins.^91^ Approximately 90% of the solvent is removed during the film shrinkage in the first half of the drying time, and the remaining 10% takes another half-time.^63^ The second half of drying, which is pore emptying, needs extensive time and energy. It has been estimated that nearly 30–55 kWh of energy is required per kWh of cell.^92^ An aqueous solvent instead of NMP can significantly accelerate drying. It reduces the time needed by a factor of 4.5 and requires 10 times less drying energy per kg of solvent.^63^ To further reduce energy consumption by 25%, the “green coater” technology is developed to reuse hot air in the dryer unit. This technology integrates the dry furnace with an organic solvent collection unit, allowing heat collection, circulation, and reuse, which previously escaped through the exhaust (developed by Toray Engineering).^93^ As drying is already energy intensive, NMP recovery systems can benefit the manufacturing industries financially. An NMP recovery system is based on a high-performance, energy-efficient dry rotor system using advanced honeycomb rotor technology. It provides environmental compliance by ensuring exhaust-free NMP recovery. The closed-loop design (by Hitachi High-Tech Corporation) prevents dry air wastage, and the system also provides a lower total cost of ownership.^94^ An adsorption wheel–based heating, ventilation, and air conditioning system is provided for recovering exhaust solvents by Taikisha USA Inc.^95^ An air circulator is patented by Dürr Systems Inc. to condense most NMP from the generated exhaust during drying.^96^

In lab-scale drying, the coating is typically dried at ambient temperatures. In industrial settings, drying is achieved using large belt dryers equipped with infrared (IR) radiators or hot air fans (convection ovens).^97^ Industry-standard drying parameters include rates of 25–50 m/min, dryer lengths of 20–60 m, and a coating residence time of 1–3 min.^98^ The drying rate is influenced by the drying temperature, which ranges from 50°C to 160°C,^99^ and the air velocity.^97^ The air flotation dryer used in industries can be as long as 70 m, with temperatures reaching up to 150°C to ensure even coating drying.^100^ In addition to the conventional drying methods, several innovative drying technologies have emerged. IR drying uses special IR emitters to generate heat. Some companies reported up to a 90% reduction in drying duration with advanced drying technologies. The drying rate by utilizing IR is twice as fast compared to conventional convective drying methods. Near-IR (NIR) requires higher energy input compared to mid-IR. However, compared to conventional drying, it can lead to an 85% reduction in energy consumption for cathode production and a 60% reduction in energy consumption for anode production.^101^ Additionally, the environmental footprint could be reduced by 2.63 million Mt CO_2_eq per year with the implementation of NIR drying technology.^102^ IR radiation is used in the compact dryer model by Hitachi High-Tech Corporation to increase drying efficiency.^103^ In laser drying, a laser beam radiates onto the slurry, which absorbs the radiation and converts it to thermal energy. Laser drying is also a more efficient and sustainable alternative for electrode manufacturing compared to traditional furnaces, as it can reduce energy consumption by 50%. Its implementation can also lower the environmental footprint by 1.47 million Mt CO_2_eq per year.^102^ This drying technique can reduce the required dryer length and lower associated costs. For example, a three-stage laser drying process using vertical-cavity surface-emitting lasers (VCSELs) can achieve complete drying within 20 s, hence resulting in a 90% reduction in drying time.^104^ In microwave drying, the electrode coating is heated by exciting polar molecules with microwave radiation.^105^ Using diode lasers has also been initiated because of their efficiency and relatively lower environmental impact than conventional convection air dryers by companies such as Laserline.^106^^,^^107^

One of the most critical challenges faced during the drying process is the redistribution of the binder, also called “binder migration,” which significantly affects the electrochemical performance of batteries by forming a binder concentration gradient.^108^ This issue arises when solvent molecules drag the polymeric binder structure during evaporation and increase the tortuosity of the battery. Adjusting the drying rate can optimize such binder migration.^97^ Furthermore, drying can cause cracks, stress, and delamination of the electrodes. To address these issues, innovations in dry electrode processing or solvent-free electrodes have been proposed through pulsed laser and sputtering deposition.^109^^,^^110^ A spray system, i.e., dry painting or electrostatic spraying, is also suggested for coating dry electrode particles onto current collectors.^109^ Considering the advantages of dry electrode processing, Tesla will use dry-coating technology to produce 4680 cylindrical cells,^111^ and LG Energy Solutions plans to commercialize its dry-coating process by 2028.^112^

### Calendering and final drying

The next manufacturing stage is calendering, where a dried electrode coating is pressed under external pressure.^69^ Out of the total battery cost, the calendering process accounts for only 5%.^113^ However, this growing market is projected to increase at a CAGR of 19.7% to $42.60 billion by 2032.^114^ The calendering process reduces the porosity of the coating layer by 20%–40%,^115^ and hence, it increases the density and electronic conductivity of the cell because of the enhanced electrode–electrode particle interaction and improved electrode–collector interaction.^116^ This step involves re-establishing particle–particle and particle–binder interactions after drying, thereby defining the final electrode structure and the related properties.^117^ Furthermore, calendering improves the adhesion of the electrode particles to the current collector and smoothens the electrode surface, thereby reducing short-circuit risks. However, finding the right balance between thickness, porosity, electron/ion transport, and mechanical properties of the electrode coating is crucial when determining the optimal calendering conditions.^69^ Calendering also affects electrolyte wettability, which can favorably improve battery performance depending on the porosity of the dried electrode coating.^118^ Nevertheless, excessive calendering can decrease the diffusion path and cause particle rupture and interface fusion, leading to parasitic side reactions and a shorter life cycle.^119^^,^^120^

The pressure applied to the dried coating during calendering is typically achieved using rollers. Hence, roller diameter, temperature, speed, and geometry significantly affect the calendering process. Temperature eases malleability, speed reduces energy consumption and processing time, and diameter and geometry affect contact area and pressure distribution.^121^ Various roller systems are available, including two-roll, three-roll, four-roll, and mult-iroll systems. Among these roller systems, the two-roll system is mainly used. In a two-roll system, two rollers with a defined gap press the dried coating to the desired thickness. Two-roll calendering machines are optimized for achieving a coating thickness of 10–150 μm and a density of 1.5–2 g/cm^3^ (anode).^122^ The rollers, usually made of steel and coated with chromium, can operate under hot or cold conditions, generally called hot or cold calendering. Hot calendering offers a higher compaction rate and lower rebound rate than cold calendering, although cold pressing is simpler.^123^ The reason for the high compaction rate in hot pressing is that at high temperatures, the distribution of the electrode particles improves, residual stress is reduced, and hence, the electrodes are densified.^98^ Owing to these advantages, high-temperature calendering options are offered by industries such as Lead Intelligent Equipment Co., Ltd., Andritz AG, and Hitachi High-Tech Corporation.^124^^,^^125^^,^^126^ An innovative production setup of the calender allows foil widths to exceed 2000 mm. This advancement was realized using an axial roll to ensure even load distribution. The system also has a roll-bending cylinder that aligns itself, with roller temperatures of up to 160°C.^124^ While the porosity and mechanical properties of the electrodes depend on the applied pressure, the conductivity depends mostly on the temperature because of the increasing deformability of PVDF with temperature. When the rolling temperature increases, the porosity and compaction resistance of the electrodes decrease, and, hence, adhesion strength is increased. However, high-temperature calendering can reduce the battery capacity and is not recommended (>120°C).^113^^,^^127^ With the growing interest in dry or solvent-free electrode processing, the design of the calendering equipment is changing to provide proper shearing for alternative material nature.^128^

After calendering, the electrode coating passes through the drying process to eliminate any moisture or solvent content that causes potential battery performance issues. Moisture causes unwanted side reactions with the electrolyte, such as the evolution of gaseous hydrogen fluoride, which reduces conductivity and degrades battery performance.^129^ Vacuum drying is typically used for the final drying stage. The vacuum lowers the boiling point of liquids, which eliminates heating and ensures safety. The Fraunhofer Research Institute for Battery Cell Production (FFB) uses vacuum dryers with exhaust air treatment for their Gigafab expansion.^130^ Roll-to-roll vacuum drying technology is offered by companies. NGK Insulators has patented slalom-configured roll-to-roll furnaces utilizing wavelength–controlled heaters. This technology enables continuous drying and eliminates the need for a drying chamber. This innovation prevents inventory buildup, reduces energy consumption by cutting the required dry air volume in half, and optimizes space efficiency. Moreover, it reduces drying time to 10 min, approximately 1/100th of the time needed for conventional vacuum batch drying.^131^

## Equipment for cell assembly

**Figure 9:**
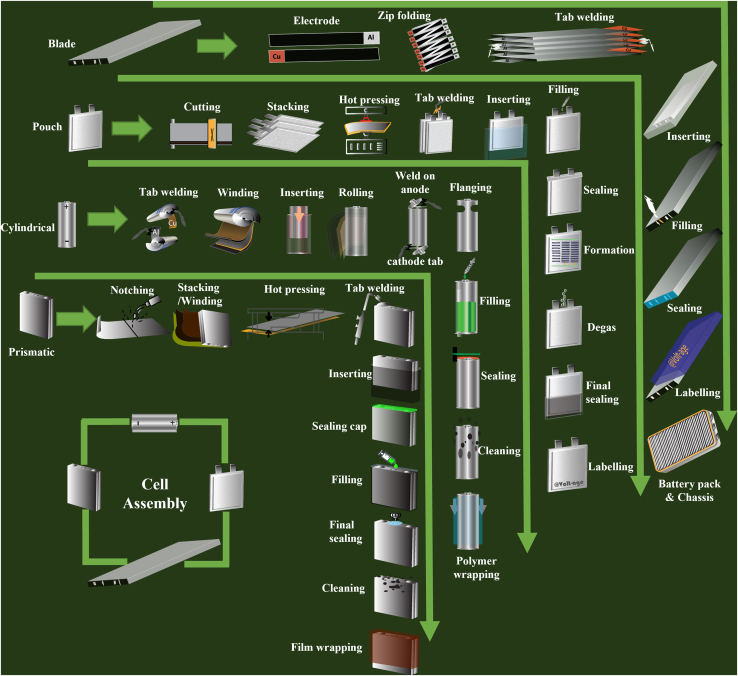
Cell assembly process at a glance based on cell design

The dried coatings will undergo the cell assembling process, which varies to some extent for different final cell shapes, e.g., winding for cylindrical cells vs. stacking for pouch cells. Figure 9 schematically shows these differences.

### Slitting and notching

Once the coated foil reaches this stage, it is much larger than that required for each cell. Thus, it needs to be cut down to the appropriate size using slitting and notching. Slitting cuts the foil to the desired shape. For cylindrical cells, the foil is cut into long, narrow strips suitable for rolling into a jelly roll. For prismatic and pouch cells, the foil is cut into a rectangular shape.^132^ This cutting process should be precise to provide battery fit and performance and generate minimum waste.^133^ The cross-sectional area of the anode is larger (approximately 2 mm) than that of the cathode because this extra space accommodates the reversible capacity loss and reduces any alignment error that might occur during the next stage of battery assembly.^98^ Several parallel blades are used to obtain several strips of the same width simultaneously during slitting.

**Figure 10:**
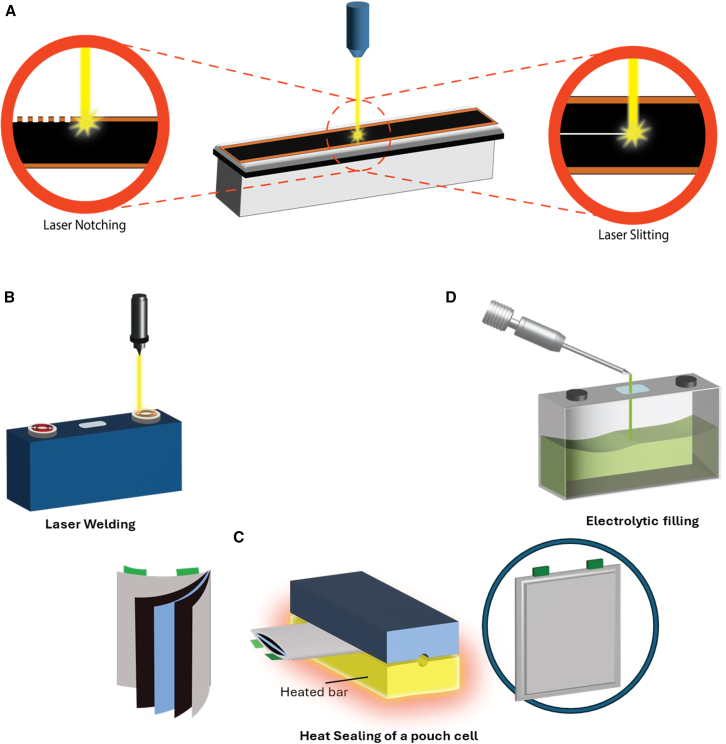
Cell assembly (A–D) (A) Laser-based slitting and notching, (B) laser welding, (C) heat sealing, and (D) electrolyte filling.

On the basis of the cell type (pouch, prismatic, and cylindrical cells), different mechanical cutting technologies are used to match the design requirements of each battery type. Rotary knives and die cutting use blades made from hardened alloy to cut the coated foils precisely. Die cutting is commonly used for pouch and prismatic electrodes, and rotary knife slitting is used for cylindrical electrodes.^134^ CATL’s battery production plant uses roller-slitting machines.^100^ Mechanical cutting technologies suffer from mechanical wear and tear and eventually produce lower-quality electrodes with slits.^135^ Some mechanical slitting machines are equipped with a specialized gang-cutting method to reduce burr formation and ensure higher-quality cuts in the slitting process, i.e., Nishimura’s TG124E and TG104E models.^136^^,^^137^ Laser cutting (Figure 10A) is being exclusively adopted because of its advantages, such as the reduced risk of burr formation, smoother edges, and lack of mechanical wear, by companies such as Manz AG.^138^ It is also more cost-effective than traditional mechanical cutting technologies. However, the laser cutting process could operate slower than the traditional cutting process, which diminishes its utilization for mass production capacity. This technique can achieve cutting speeds ranging from 25 to 50 m/min.^139^ Using laser technology for electrode cutting consumes less energy due to its highly concentrated laser beam, which enhances efficiency while reducing overall energy consumption and carbon emissions.^140^ IR laser can achieve a cutting speed of up to 30 m/min while consuming only 54W of power. A notable challenge with conventional lasers is their longer pulse durations, which can create heat zones on the cut edges and adversely affect the electrode material properties. This issue can be mitigated using ultrashort laser pulses measured in femtoseconds.^141^

Another issue in the cutting process is related to the edge quality. Ideally, these machines should have a slitting error tolerance of 0.1 mm and a rewinding speed of 100 m/min and should consistently produce high-quality products. Researchers and industries focus on developing state-of-the-art technology that has burr edges below 10 μm.^133^ In addition, dust generated by slitting the coated electrodes may negatively affect electrochemical performance and may cause short circuits if deposited on the electrode surface. To address this issue, a vacuum suction system is used to continuously discharge waste and prevent disruptions in the manufacturing process.^142^ In addition to vacuums, using brushes and dedusting fans are other solutions to overcome this issue. A flow-optimized extraction and supply air laser chamber is patented by Manz AG with a novel design to remove contaminants from the end product.^138^

In the second cutting stage, i.e., notching, the electrode foil’s outer edges are trimmed into rectangular tabs (Figure 10A). This process uses technologies similar to slitting, such as die cutting and laser cutting. Laser notching offers the advantage of greater flexibility in the cell formats and tab geometries. The laser progressive notching technology customizes the lengths of electrodes and distances between them. Laser technology is highly adaptable to innovative designs, such as the patented “tabless design” by Tesla.^138^^,^^143^

### Stacking/winding

Cell type (cylindrical, prismatic, pouch, or blade) directly impacts the choice of stacking or winding. Cylindrical cells are assembled using the winding process, while prismatic cells can be assembled using both stacking and winding methods. Pouch cells mainly utilize the stacking process, although the winding process can also be applied in certain designs. Blade cells, on the other hand, can only be assembled using the stacking process.

### Winding

**Figure 11:**
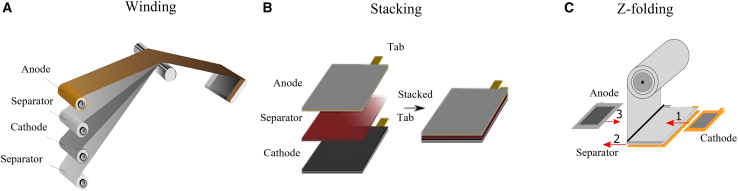
Assembly methods (A–C) Assembly methods: (A) winding, (B) stacking, and (C) Z-folding techniques.

Winding machines use tension reducers, unwinding mechanisms, and three-position winding.^144^ Large rolls of electrode materials and the separator are carefully unwound and fed into the winding machine (Figure 11A). In the machine, layers of anode, cathode, and separator are aligned with high precision and tightly wound into the desired configuration to avoid misalignment causing increased risk of short circuits.^145^ In cylindrical cells, the jelly roll tightly winds the layers in a spiral formation. This process minimizes interlayer spacing for uniform electrolyte contact and consistent electrochemical performance.^146^ In prismatic cells, the electrodes are wound in a flat or oval compact configuration to improve energy density and make them flexible and suitable for various applications.^147^ The jelly roll winding process is widely used for high efficiency in fast production and for mechanical stability. However, this process presents significant challenges in thermal management due to the limited surface area available for heat dissipation (specifically in cylindrical cells), a critical factor in high-power applications such as EVs.^148^^,^^149^ Tension variation causes uneven winding, wrinkles, or gaps between layers, which deteriorate the cell’s mechanical stability and safety.^150^

A tabless design is advanced, particularly with the 4680 formats (4.6 cm in diameter and 8 cm in length), that improves current distribution and thermal management to make these highly efficient for EVs (employed by Tesla and Panasonic).^151^ High-precision winding machines are produced with the aid of automated tension control and alignment sensors for consistent winding quality and tightness (Kaido (Japan)^152^ and Xiamen Tmax Battery Equipments Ltd.^153^). These machines facilitate the precise winding of cylindrical cell electrodes to reduce defects and optimize performance during mass production. Semi-automatic winder for cylindrical and prismatic cells integrates programmable winding patterns and precision tension control. The machines can be implemented for consistent electrode winding even for larger cells like the 46100 formats. Thus, it can enhance the uniformity in the winding process and reduce misalignment, enhancing battery performance.^154^

Winding machines with real-time alignment monitoring and automated tension regulation is offered. This would increase the precision required for the production of high-quality cylindrical cells and maintain electrode alignment and proper tension throughout the winding process.^155^ Some winding machines can quickly adapt to different cell formats and enhance the flexibility of the manufacturing process.^156^ A typical winding machine with a diaphragm precisely aligns the layers with ±0.3 mm accuracy.^157^ Laser technology helps more efficient and reliable winding by proposing high-speed, precise laser cutting that ensures clean edges and minimizes contamination.^158^^,^^159^

Strain gauges during winding offer monitoring of mechanical stress and deformation and prevent defects.^160^ Uniform tension is crucial to avoid uneven winding and defects, such as wrinkles and microcracks, utilized by CATL and LG Energy Solution. A range of tension controllers consists of load cells, tension indicators, and controllers that integrate into the winding equipment to maintain consistent tension.^161^ Optical micrometry measures electrode and separator thickness to ensure uniformity during winding and prevent internal short circuits. Keyence offers optical micrometers and laser displacement sensors for material thickness measurements in the winding line.^162^ Thermal cameras, such as forward-looking infrared (FLIR), monitor temperature distribution across the wound cell to detect hotspots and prevent material damage.^163^ With acoustic emission sensors, stress waves emitted by materials can be detected during winding. This method identifies local stress or material fatigue that leads to defects.^164^ The segmentation and bending of uncoated electrode portions could enhance flexibility and precision during the winding stage. This innovation helps to improve the current collection, reduce resistance, and minimize mechanical deformation, which is critical in cylindrical battery winding.^165^ To improve winding precision and reduce cell defects, a variety of advanced sensing and monitoring methods have been implemented in LIB manufacturing. Implementing advanced taper tension profiles has been shown to reduce maximum tension errors by approximately 16.1% during the winding process, which results in fewer mechanical defects.^150^

After winding, nondestructive inspection and imaging technologies analyze cell quality without damage. Advanced monitoring techniques like X-ray computed tomography (CT) imaging are critical for battery cell inspection. CT scanners, by primary investigation of the samples, provide high-resolution 3D images for the non-destructive analysis of a cell’s internal structure after the winding process to detect issues such as misalignments and voids.^166^

### Stacking

Stacking is layering anode, separator, and cathode sheets in a compressed, sandwich-like structure to enhance packing efficiency (Figure 11B). A rectangular stacking format in prismatic cells optimizes space utilization and enhances volumetric energy density compared with cylindrical cells. It allows the precise alignment of electrodes and results in a more efficient packing of AMs and better energy storage.^167^ Although maintaining uniform pressure across layers is challenging, it is crucial for preventing delamination and ensuring long-term reliability.^168^ Electrode materials are susceptible to mechanical damage during stacking and compression, with inadequate or excessive compression leading to defects.^169^

Stacking pouch cells is more complicated because of electrode flexibility, which causes wrinkling, misalignment, and uneven pressure, especially in bigger stacks. Typical modular equipment cuts electrodes precisely places them between separators and laminates them under heat and pressure.^170^ Lamination is utilized to enhance energy density and mechanical stability.^171^ Vacuum-assisted stacking secures flexible electrodes and separators during assembly with accurate positioning and avoids distortions. This technique leads to uniform compression, improved energy density, and extended cycle life.^172^ The Z-folding stacking machine creates a stack or jelly roll. This lamination stacking machine performs multiple functions of unwinding, punching, marking, lamination, stacking, taping, and unloading. This machine is designed with constant speed and web tension control, high-precision CCD defect inspection, and high-speed laser marking for optimal production quality.^173^

Stacking prismatic cells requires precise alignment of electrode and separator layers, which is complex and costly, especially in large-format cells. Another technique is Z-folding with advanced folding in a zigzag pattern (Figure 11C) to improve layer alignment and speed up stacking with fewer defects and higher reliability.^117^ The Gen 3 Z-folding equipment adopted by SK Innovation demonstrated a 2.3× increase in speed compared to the Gen 1 used in 2010 to enable faster throughput and reduce machine demand per production volume.^174^

Advanced stacking technologies can switch between Z-fold and single-sheet stacking based on specific requirements, which makes the process more suitable for applications such as solid-state batteries.^175^ Blade cells undergo a lamination process. However, the lamination process for ultra-thin, elongated electrodes in blade cells presents challenges, especially in maintaining uniform compression across electrodes and consistent electrolyte distribution. The thin electrodes might also be more susceptible to mechanical damage during stacking.^169^

High-resolution machine vision systems, such as Stemmer Imaging, have been used for optical inspections during the stacking process to verify the precise alignment of electrodes and separators.^176^ Peel testers measure the adhesion strength between layers during stacking. This is critical for prismatic and blade cells, where strong adhesion helps in maintaining the cell’s structural uniformity.^177^^,^^178^ Continuous thickness and consistency measurement in specified tolerances is obtained by utilizing caliper sensors.^179^ IR thermography for heat generation monitoring is particularly useful during the stacking of the blade cells. For instance, thermal cameras by FLIR monitor heat generation to identify hotspots or thermal inhomogeneity.^180^

Insulation resistance testing is conducted after stacking or before electrolyte filling to ensure proper separator insulation to prevent short circuits and electrical failures.^181^ Compression test using universal testing machines is conducted after stacking to ensure uniform pressure across the cell stack, which is essential for optimizing the mechanical integrity of the battery components.^182^ Some companies provide material testing machines and structural testing.^183^^,^^184^ Swelling and thickness measurements using laser sensors are also taken after stacking to evaluate the layer’s uniform thickness and prevent mechanical stress during charge–discharge cycles, particularly in pouch cells.^185^ Partial discharge testing is mainly used after stacking to detect defects in the insulation materials by identifying localized dielectric breakdowns as early indicators of potential faults.^186^

Once the stacking process is completed, the stacked cell layers are inserted into a casing, which can be a rigid metal or plastic case for prismatic cells, a flexible pouch for pouch cells, or a blade structure for blade cells. Then, cells undergo electrolyte filling, where the electrolyte is carefully injected into the cells. It will be sealed and prepared for formation.^187^

### Equipment for cell finishing

Different cell formats will undergo a specific cell finishing process (Figure 10). Welding, sealing, and filling are performed in different orders. Welding is performed once or repeatedly on various spots in the cell, i.e., on anode and cathode tabs in cylindrical cells. Sealing is also performed partially in different steps to seal the cell fully.

### Welding

The stacked or wound cells are transferred to a dry room, along with dried separators for cell assembly. Aluminum, nickel, or copper tabs are then attached to the cathode and anode current collectors. Although welding accounts for a small portion of manufacturing costs and energy consumption (7.34% and less than 2%, respectively) and is highly automated, challenges in cell tab joining persist. These unresolved issues can lead to significant safety concerns. The high operating temperatures of LIBs, particularly in automotive applications (up to 80°C), can increase connection resistance and can cause temperature fluctuations. This may result in thermal expansion or fatigue, which potentially damages the tab joint.^188^ The quality of a weld is significantly influenced by contact resistance. When this resistance is high, issues such as energy inefficiency and excessive heat generation occur. This heat can be detrimental by causing the degradation of cells or, in more severe cases, triggering thermal runaway.^189^ The choice of welding technique is influenced by how battery cells are packaged. Common joining methods include ultrasonic welding, wire bonding, force fitting or mechanical assembly, soldering and brazing, laser and resistance spot welding, friction stir welding, tungsten inert gas welding, joining through forming, and adhesive bonding. Ultrasonic welding is a popular choice for connecting pouch cell tabs and is sometimes used for cylindrical and prismatic cells.^190^

Ultrasonic welding, with low energy consumption and the ability to join dissimilar materials, has limitations in terms of joint thickness and potential high heat generation. By contrast, resistance welding is cost-effective and requires low thermal input for joining cylindrical and prismatic cells. However, this welding has challenges in joining highly conductive materials or dissimilar substances.^191^ Laser beam welding (Figure 10B) is the most suitable method for battery welding because it provides the lowest electrical contact resistance and highest joint strength.^192^ Nonmetallic materials, such as plastics, can also be welded using laser welding, which allows various materials to be utilized for cell assembly.^193^ In addition, laser welding can result in a precise and narrow weld line, which can lower the cell’s weight and size and increase its energy density.^194^ During all welding procedures, the generated heat must be concentrated and must not reach levels that damage the cell.^195^ Decreasing the wavelength and shortening the pulse width to the nanoscale are offered as a solution.^196^ Advanced welding machines include lid laser welding machines, collector laser welding machines, ultrasonic welding machines, cap laser welding machines, anode welding machines, and tab welding machines. Each offers specialized functionalities such as high-speed ring spot welding, CCD detection, and real-time quality inspection.^173^ Manz AG offers laser technologies of overlap welding, zero-gap welding, and bimetal welding.^197^ Manz AG declares that its innovative laser tab welding eliminates one process step compared to traditional ultrasonic welding. This reduction in complexity leads to significant cost savings, with the investment in laser technology proving worthwhile in less than a year due to decreased operating costs and increased throughput.^198^

### Sealing

Vacuum sealing of the Li-ion cells is to safely enclose them, shield them from moisture and contaminants, and strengthen them structurally for stability in the long run.^199^ Partial sealing is performed before filling, and complete sealing is performed after filling to seal the opened sites (filling side). A precise and long-lasting final seal is essential to preventing environmental exposure. Vacuum sealing machines,^200^ play a crucial role in encapsulation with precise airtight seals. Heat sealing, hot air welding, ultrasonic welding, and chemical adhesives are examples of sealing technologies. The heat-sealing technique (Figure 10C) is the most widely used, where thermoplastic films with a thickness of less than 0.5 mm are joined. Hot-bar welding and impulse welding are the two forms of heat sealing.^201^ High-precision laser welding technologies are developed by providing stronger, more reliable battery cell seals.^202^ A challenge in sealing is maintaining consistent airtight seals because imperfections can lead to contamination. Obtaining strong and consistent bonds during the battery sealing and welding process is complicated because weak points can develop because of inconsistent material properties (such as variations in electrode coatings or separator thickness) or irregularities in the welding process (such as uneven heat application or pressure).^203^

### Filling

Before the initial charge and formation process, the electrolyte must fully saturate all cavities and pores in the anode, cathode, and separator in the filling process (Figure 10D).^204^ This step is time-consuming and costly, typically 12–24 h in a dry room. Various factors, such as elevated temperatures and electrode and cell assembly specifics, affect the wetting process.^205^ Wetting is defined as the ratio of the electrolyte infiltrating the cell stack’s pores to the total available pore volume.^206^ A varying pressure profile is applied to the cell during and after filling to activate the capillary action of the porous electrode, ensuring effective electrolyte wetting.^104^ Inadequate filling and wetting of the electrolyte can cause issues that affect the cell’s performance, lifespan, and safety. In particular, if the electrolyte fails to penetrate the electrode pores fully and uniformly wet the active particles, the solid electrolyte interphase (SEI) layer might not form uniformly on the active particles during the initial cycle.^207^ This can result in electrolyte breakdown during use, reduced Coulombic efficiency, or the initiation of lithium dendrite growth.^208^ Several dosing steps may be required to fill the entire electrolyte volume into the cell, especially for large prismatic or cylindric cells, which results in process times of up to three weeks.^209^ The facilities and handling steps required for dosing, wetting, and forming cells represent an additional cost factor during large-scale LIB production.^209^ Wetting time reduction decreases the overall production costs, commonly performed by inducing a lower-than-atmospheric pressure in the cell, e.g., 100 mbar.^210^ Fully automatic electrolyte-filling machines provide vacuum filling, soaking, and vacuum heat sealing.^173^ Vacuum sealing is performed to close the cells completely and to fulfill the safety criteria. The sealed cells are washed to remove the remaining electrolyte.

### Formation

**Figure 12:**
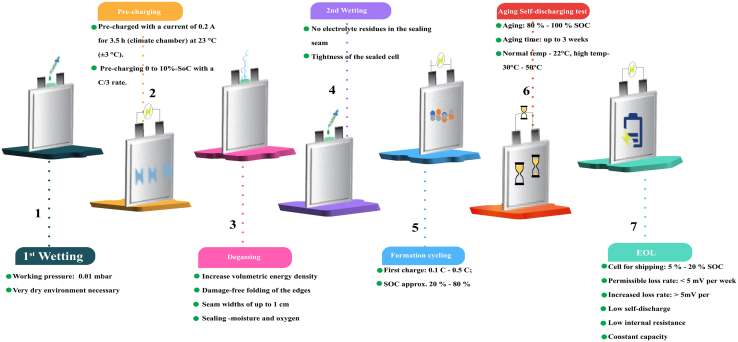
Schematic overview of the cell formation procedure

Throughout this process, the protective SEI layer forms on the surface of the anode’s AM.^211^ Figure 12 shows a schematic view of the formation process. To ensure a stable SEI layer on the anode’s surface, the cells are charged/discharged at a low rate, such as C/20, and then the rate is gradually increased. It typically takes several low-rate charge and discharge formation cycles to produce a dense and stable SEI layer.^212^ The gas produced during the formation process must be released for safety reasons. The cells are kept on the aging shelves for full electrolyte wetting and SEI stabilization during or after formation cycles.^213^ Cell formation is completed by aging, where the cells are stored at a specific temperature and state of charge. It also functions as a self-discharge test. Given that a higher capacity loss during storage indicates undesired self-discharge, aging can be viewed as a component of the EOL cell quality test.^214^

Aging and cell formation accounted for 33% of the total manufacturing costs or $30 million annually (Figure 1A).^24^ Improving productivity and cutting costs in LIB production require reducing the electrolyte wetting, formation, and aging times. These procedures substantially add to manufacturing costs but are necessary to guarantee consistent capacity, long cycle life, and high-quality LIBs. Aging can take up to two weeks, and wetting and formation can take three to seven days. When taken entirely, these procedures account for up to 25% of the production floor area and a significant amount of plant capital expenses.^213^ To reduce energy consumption and promote greener battery production, new formation equipment has been developed with a recuperation feature that uses the discharge energy from one cell to charge another. Managing and precisely controlling power delivery to formation equipment for an entire gigafactory present a significant challenge. A recent innovation aimed at cutting the cost of the formation process by designing it to process groups of cells together rather than individually, leading to an 86% reduction in formation investment costs.^215^ Another report estimated a decrease in the formation and aging time of 60–70% by 2030. At the same course of time, a reduction of the percentage of the rejected cells from 15% to 1% during final quality control would also be promising in the capital expenditure costs.^216^

To finalize the assembly and finishing, Li-ion cells are packaged precisely to ensure their longevity and safety when assembled into modules or packs.^217^

## Conclusion and perspectives

### Conclusion

Significant research in battery manufacturing is being conducted on optimizing material selection, electrode preparation, and assembly processes, while the critical role of manufacturing equipment remains underestimated. Advancing cell manufacturing processes, especially regarding cost efficiency, time reduction, and overall productivity, heavily depends on innovative machinery and equipment. Giant equipment manufacturers not only build a large portion of the required equipment but also take the lead for innovations and cost-effective production. Hence, battery plants significantly depend on them, with expertise spanning the entire production line. Nevertheless, this dominance raises concerns regarding the global supply chain’s resilience and sustainability. By identifying the major players in the equipment manufacturing landscape and outlining their challenges, we tried to give an industrial perspective on the current machinery available for battery production. We aimed to provide academic researchers and industrial partners with a clear understanding of the present situation and its drawbacks. By focusing on new solutions and pathways in cell manufacturing equipment, we hope to inspire greater collaboration between academia and industry to enhance production efficiency, reduce costs, and advance the development of next-generation batteries.

### Perspective

To address these concerns and increase sustainability in equipment manufacturing, we presented some perspectives for those who are interested:

- Regions must support OEMs to reduce their dependency on specific companies. Equipment shipment, training, and maintenance are significantly delayed. Gigafactories often wait long for critical equipment. For example, a battery plant in North America might wait years for coating machines and assembly lines from China. Equipment manufacturing must be localized to reduce lead times and maintenance response times. The machinery can also be customized to meet specific local requirements, such as more demanding European environmental standards. Local machine shops will implement industrialization with new designs and basic intellectual properties for zero waste and highly automatic speed processes.

- Equipment’s functions and their specific challenges and limitations must be analyzed to meet precision, efficiency, cost, and scalability requirements. Some solutions are the uniform distribution of materials in mixing and coating, online examinations, double-side coatings, and Z-folding. While developing an NMP recovery system offers significant benefits, dry electrode coating processes eliminate the need for drying in traditional wet electrode manufacturing, with a substantial reduction in energy consumption for large-scale drying in gigafactories. Laser technology for slitting, notching, and welding offers significant advantages over traditional mechanical tools, including higher precision, increased speed, and reduced material wear.

- Another priority will be creating space-saving equipment that combines several processes into one machine to improve efficiency and cut costs at gigafactories and battery plants. In the past, cells were charged and discharged individually, requiring large spaces and energy. More compact, multifunctional machines can transform battery production, making it more efficient in terms of space, cost, and scalability. The future of battery manufacturing will see increased integration of space-saving equipment and advanced formation processes that reduce production time and costs. Innovations such as simultaneous cell formation processes, seen in companies like Tesla and Panasonic, exemplify how global manufacturers are optimizing battery production lines to meet the demands of electrification and sustainable energy storage worldwide.

- Equipment manufacturing can rely on green production. Enormous sustainable energy sources can supply gigafactories with minimum environmental impacts, such as green hydroelectricity, with traceability, certification, and passport. In addition, green mines in places such as Finland and Canada ensure a sustainable supply of critical raw materials such as lithium, cobalt, and nickel. South America, particularly Chile, is focusing on sustainable mining practices for lithium extraction, ensuring a cleaner supply chain for the global battery industry. Companies in Asia, such as CATL, are pioneering solvent recovery and recycling technologies, while Northvolt in Sweden is utilizing 100% renewable energy for its gigafactory operations. In Africa, local manufacturers are exploring ways to utilize abundant solar energy to power battery production facilities. In addition, the logistical challenges in equipment transportation will be reduced by utilizing local equipment. These effects reduce carbon emissions and support sustainable battery manufacturing ecosystems. Sustainability in battery manufacturing is not limited to any region but is a universal goal. Across the globe, from Asia to Europe and the Americas, manufacturers are adopting green energy, solvent-free processes, and recycling technologies to minimize environmental impact.

- One promising approach for successive equipment manufacturing is to form joint ventures and partnerships among smaller OEMs. Small companies can produce all necessary machinery for battery plants by combining resources and expertise. Local manufacturers will scale up and cover the entire machinery for a battery plant through collaborations, from producing electrodes to the final cell formation. Localizing innovation and equipment manufacturing will build a sustainable and competitive battery manufacturing system. With strategic planning and proper investment in partnerships, regions will reduce their reliance on specific manufacturers and turn to key players in the global battery market for the widespread adoption of electrification. By fostering partnerships between OEMs and research institutions across Asia, Europe, North America, and emerging markets, regions can reduce vulnerabilities in the equipment supply chain and support the development of more sustainable production technologies.

- Battery technology is changing with the introduction of new chemistries and technologies such as solid-state systems or sodium-ion batteries. Hence, manufacturing machines must accommodate these innovations easily and quickly. For example, precise handling of solid electrolytes must be considered in solid-state batteries to fit the characteristics of new electrode materials. Sodium-ion batteries with varying chemical properties from lithium-ion batteries need mixing, coating, and formation modifications. This means that future manufacturing equipment must be designed for high efficiency by considering these advancements. Therefore, gigafactories can easily adjust to new battery chemistries without entirely new equipment, which saves on costs and maintains production scalability.

## Resource availability

### Lead contact

Requests for further information and resources should be directed to and will be fulfilled by the lead contact, Karim Zaghib (karim.zaghib@concordia.ca)

### Materials availability

The figures’ data will be available on request.

### Data and code availability

This paper does not report original code.

## Acknowledgments

Funding: This review paper was made possible through the generous financial support of 10.13039/501100002914Concordia University, AI–Mogul, Innovéé (Quebec Government), and 10.13039/501100000038Natural Sciences and Engineering Research Council of Canada (NSERC).

## Author contributions

All the authors participated in writing and revising the paper. K.Z. supervised the team.

## Declaration of interests

The authors declare no competing interests.
